# Correlation between Plasmonic and Thermal Properties of Metallic Nanoparticles

**DOI:** 10.3390/nano14100820

**Published:** 2024-05-07

**Authors:** Inès Abid, Javier González-Colsa, Christophe Naveaux, Andreea Campu, Célia Arib, Monica Focsan, Pablo Albella, Mathieu Edely, Marc Lamy de La Chapelle

**Affiliations:** 1 Institut des Molécules et Matériaux du Mans (IMMM-UMR CNRS 6283), Université du Mans, Avenue Olivier Messiaen, 72085 Le Mans CEDEX 9, France; inesabid24@gmail.com (I.A.); christophe.naveaux.etu@univ-lemans.fr (C.N.); celia.arib@univ-lemans.fr (C.A.); mathieu.edely@univ-lemans.fr (M.E.); 2 Group of Optics, Department of Applied Physics, University of Cantabria, 39005 Santander, Spain; javier.gonzalezcolsa@unican.es (J.G.-C.); pablo.albella@unican.es (P.A.); 3 Nanobiophotonics and Laser Microspectroscopy Center, Interdisciplinary Research Institute in Bio-Nano-Sciences, Babes-Bolyai University, Treboniu Laurian 42, 400271 Cluj-Napoca, Romania; andreea.campu@gmail.com (A.C.); monica.iosin@ubbcluj.ro (M.F.); 4 Biomolecular Physics Department, Faculty of Physics, Babes-Bolyai University, Mihail Kogalniceanu No. 1, 400084 Cluj-Napoca, Romania

**Keywords:** gold nanoparticles, plasmonics, thermoplasmonics, thermal effect

## Abstract

Here, we investigate the correlation between the heat generated by gold nanoparticles, in particular nanospheres and nanobipyramids, and their plasmonic response manifested by the presence of Localized Surface Plasmon Resonances (LSPRs). Using a tunable laser and a thermal camera, we measure the temperature increase induced by colloidal nanoparticles in an aqueous solution as a function of the excitation wavelength in the optical regime. We demonstrate that the photothermal performances of the nanoparticles are strongly related not only to their plasmonic properties but also to the size and shape of the nanoparticles. The contribution of the longitudinal and transversal modes in gold nanobipyramids is also analyzed in terms of heat generation. These results will guide us to design appropriate nanoparticles to act as efficient heat nanosources.

## 1. Introduction

Metallic nanoparticles (NPs) are increasingly gaining attention within the scientific community [1,2] thanks to their ability to confine energy in the nanoscale. These NPs exhibit a distinctive form of light–matter interaction, which arises from their capability to show Localized Plasmon Resonances (LSPRs). These are collective oscillations of their conduction electrons when they are optically excited at certain wavelengths. The excitation energy is employed in radiative (light scattering) and non-radiative (light absorption) processes. The latter, in particular, is responsible for heat generation and can be transferred from the NPs to the surrounding environment [3]. Hence, plasmonic NPs can be used as heat nanosources, offering the possibility of a high degree of temperature control at the nanoscale by simply using light [4,5].

This feature is widely used in drug delivery [6,7], nanosurgery [8,9], photothermal imaging [10,11], optimizing solar cell efficiency [12], and in cancer therapy [13,14,15]. Among cutting-edge cancer treatment technologies, such as immunotherapy, gene therapy, and targeted therapies, plasmonic photothermal therapy (PTT) is a promising pathway. Plasmonic PTT exploits the optical properties of NPs to selectively target and destroy cancer cells. Metal NPs, often made of gold, are specifically engineered to target cancer cells. These NPs are typically injected into the bloodstream or directly into the tumor site. Once the NPs accumulate in the tumor tissue they are exposed to near-infrared (NIR) light so that it can penetrate the tissue deeply without causing significant damage to healthy cells. As the NPs absorb light, LSPRs are induced, converting the optical energy into heat through non-radiative processes. This localized heating raises the temperature of the surrounding tissue, destroying the targeted cancer cells. Hirsh et al. were the first to demonstrate the potential of using plasmonic PTT on tumor tissue in vitro and in vivo, by means of silica-gold nanoshells that were able to support LSPRs in the infrared. Since then, several groups have studied and developed different types of biocompatible NPs (different in shapes, sizes, or materials) to combat cancer-related diseases [16] as well as antibiotic-resistant bacteria [17].

Each of these applications requires specific properties of the plasmonic NPs. For example, NPs with strong absorption efficiency are the most suitable for PTT [18], as they offer a better light-to-heat conversion effectiveness. Conversely, in the case of photovoltaic solar cells, using NPs with high scattering efficiency is more convenient due to their ability to enhance photon scattering, hence reducing heat losses [19].

Therefore, a systematic photothermal study of different plasmonic NPs needs to be performed to find the most appropriate nanostructure in terms of light-to-heat conversion for each desired application. Such a study must be performed with regard to nanostructure composition and geometry (size and shape). Previous works have established the fundamentals of this phenomenon and its close relationship with the optical response of plasmonic structures. Thus, optical absorption was identified as the main parameter responsible for thermal generation at the nanoscale [3]. Several studies have attempted to link absorption with local heating induced by different types of NPs, such as spheres, rods, nanoshells, nano-urchins, or nanocages [5,20,21,22]. On the other hand, the heat generation of NPs in solution and in vivo has been experimentally measured [23,24,25] for different excitation wavelengths. In most of these studies, the excitation wavelength was fixed while the position of the LSPR was tuned according to the geometrical parameters of the NPs. It was found that the highest temperature increase was not necessarily obtained at the LSPR spectral position [22]. Indeed, both light absorption and scattering contribute to the LSPR, whereas the temperature increase is only related to the absorption. Thus, the maximum value of the plasmon band does not necessarily match the maximum absorption [18,26,27], inducing a shift between the maximum temperature increase and the LSPR position. To illustrate this, it is necessary to take another approach by studying the thermal efficiency of NPs as a function of the excitation wavelength. In this case, we propose to study the evolution of the temperature increase for the same plasmon resonance as a function of the excitation wavelength. This approach allows us to finely tune the excitation of the LSPR and to accurately assess the influence of the extinction intensity on the photothermal response of the NPs. We are then able to determine their effectiveness on the overall heating of the solution. Additionally, this strategy should demonstrate whether the entire plasmonic spectrum is equally efficient in terms of local heat generation or not.

In this study, we aim to further investigate the correlation between the plasmonic optical response and the heat generation induced by NPs with two different shapes, nanospheres and nano-bipyramids, by using a tunable femtosecond laser with wavelengths from 500 to 750 nm. Furthermore, the study of bipyramids is particularly significant since there are few reports on thermoplasmonics involving this type of nanoparticles in the existing literature [25,28,29,30]. Therefore, we also focus on the correlation of the thermal effects with the LSPR properties in bipyramids, paying attention to the influence of the two resonant modes (longitudinal and transversal) on the temperature increase.

## 2. Materials and Methods

**Figure 1:**
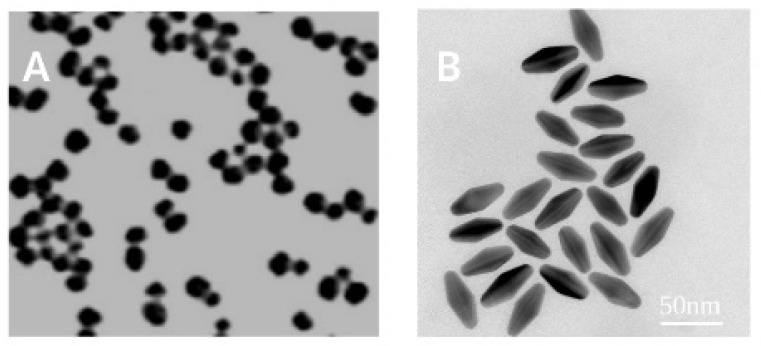
(**A**) TEM image of AuNSs with a diameter of 50 nm synthesized by Nanocs; extracted from their website [31]. (**B**) TEM images of solution of AuBPs (width around 20 nm and length around 45 nm).

Water solutions of commercial gold nanospheres (AuNSs) and gold bipyramids (AuBPs) (Figure 1) were used in this study. The colloidal solutions of AuNSs were purchased from Nanocs (Boston, MA, USA). We utilized two diameters: 50 and 100 nm. The concentrations for each solution were 9 × 10^9^ NPs/mL (14.9 pM) and 4.4 × 10^9^ NPs/mL (7.3 pM), respectively.

On the other hand, AuBPs were fabricated by using the following seed-mediated growth method [25]. We first prepared a seed solution consisting of 1 M Au salt mixed with an aqueous 25 weight % CTAC solution under vigorous stirring. Then, 0.25 M HNO_3_ and 50 mM NaBH_4_ were added as reducing agents. The final seed solution was heated to 80 °C for 60 to 90 min, after which 1 M citric acid was added. To grow AuBPs, 140 μL of the as-synthesized seeds was added to a growth solution containing 25 mM of HAuCl_4_, 45 mM CTAB stabilizing agent solution, 5 mM AgNO_3,_ and 0.4 M HQL as a reducing agent [25]. After a thermal treatment for 50 min at 45 °C, we obtained AuBPs (concentration of 116pM) with a longitudinal LSPR band at 655 nm with a width around 20 nm and a length around 45 nm as determined by analyzing the TEM images using the commercial ImageJ toolkit.

**Figure 2:**
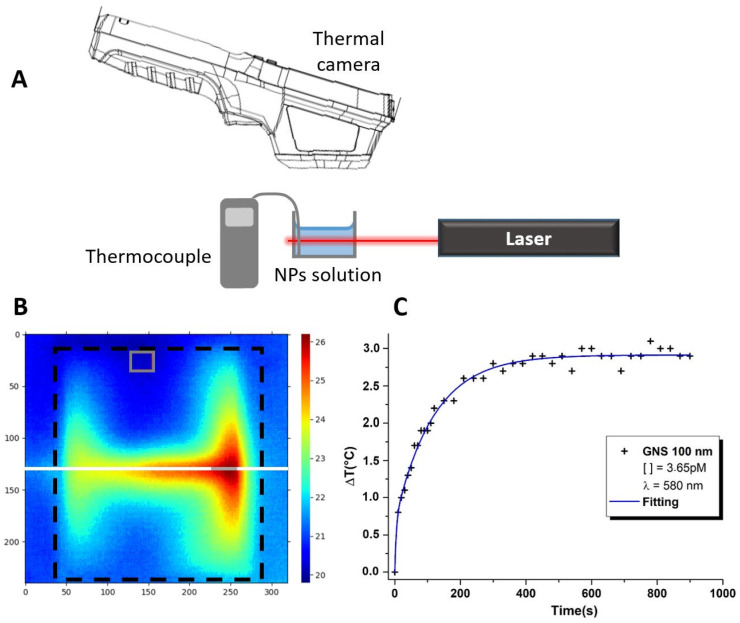
(**A**) The experimental setup scheme used to perform the photothermal measurements. (**B**) Typical thermal image of the gold NPs/water solution under irradiation taken with the thermographic camera. The black dotted square corresponds to the borders of the vessel containing the NP solution. The white line corresponds to the laser emission axis. The grey box on the top and the grey line on the laser correspond to the areas of measurements of the reference temperature and of the temperature increase, respectively. (**C**) Heating variation, obtained for the 100 nm nanospheres excited at 580 nm, as a function of the time (black crosses); the continuous blue line is the fit of the experimental data using Equation (1) below.

We used a tunable femtosecond laser (Inspire HF100 from Radiantis pumped by a MaiTai HP from Spectra Physics, pulse duration: 200 fs, 500–750 nm) to excite the NP solutions and to measure the dependence of the temperature increase on the irradiation wavelength in the NPs solutions. The laser beam illuminates the NP solution without any focusing, as shown in Figure 2 and Figure S1 (white line in Figure 2B). The laser has an output beam diameter of 1.27 mm with a divergence of 0.7 mrad. As the sample is positioned at 50 cm from the laser, the beam diameter within the solution can then be considered to be 2 mm, given the negligible divergence throughout. The laser power is set at 200 mW for all wavelengths, except when studying the dependence of light-to-heat conversion efficiency on varying laser powers. Therefore, the input power density is $I\approx 6.4\times 10^{-5}$ mW/μm^2^ ($6.4\times 10^{-5}$ W·cm^2^).

To measure the temperature increase induced by the irradiation, we used a thermal camera (TiR32 from Fluke) placed above the NP solution at a distance of 10 cm (see Figure 2 and Figure S1). The thermal camera is composed of 320 × 240 sensors offering a spatial resolution (Instantaneous Field of View, IFOV) of 1.25 mrad, with a thermal sensitivity (Noise Equivalent Temperature Difference, NETD) of 0.045 °C and a temperature measurement accuracy of ±0.1 °C. We also used a thermocouple to measure the temperature at the reference point with greater accuracy. The thermocouple position is identified by the grey box in Figure 2B.

A typical thermal image is presented in Figure 2B, where the color bar refers to the temperatures. The hottest area (red central region) corresponds to the laser beam path (reference white line in Figure 2B) inside the sample. A cone of temperature gradient is formed around the reference line displaying the heat transfer inside the solution. To determine the temperature increase induced by the NPs, we measured two different temperatures. First, we calculated the mean temperature along the grey line (Figure 2B), which corresponds to the temperature increase induced by the NPs directly excited by the laser beam (white line). A second mean temperature, which serves as the reference temperature, was calculated away from the laser beam, and is shown in the grey rectangle (Figure 2B). The temperature variation was then calculated by subtracting these two temperatures. Note that the measured temperature is the temperature of the solution and not the temperature of the NPs. The observed thermodynamic process starts with the absorption of the optical energy, which is converted into heat via the resistive losses within the gold NPs. The conversion of the optical energy into heat induced by NPs is the result of four successive physical processes occurring on time scales ranging from fs to ns: (1) the plasmon excitation and the electron oscillation (1–10 fs), (2) the energy redistribution within the NPs induced by electron-electron collisions (1–100 fs), (3) the energy relaxation during electron-phonon interactions (100 fs–1 ps), and, finally, (4) the transfer of thermal energy to the interface with the surrounding medium via phonon–phonon interactions (10 ps–10 ns) [3,32]. The heat is then dissipated into the surrounding fluid, raising its temperature. So, the higher the temperature of the NPs, the higher the solution temperature becomes. This results in greater heat generation of the solution. Consequently, different temperatures correspond to different light–NP interactions and, implicitly, to different heat generations within the metal. The evolution of the temperature with the experimental conditions (specifically, laser power and wavelength, plasmon resonance position, NP concentration, etc.) provides information on the heat generation inside the NPs and, consequently, on their thermal properties.

For each excitation wavelength, the sample temperature increase was tracked over an exposure time of 15 min. During this time, 39 thermal images were recorded: every 10 s for the first 2 min, then every 30 s for the remaining time. As an example, Figure 2C shows the temperature evolution recorded for a solution of 100 nm nanospheres that was irradiated with a 580 nm laser beam, as a function of the time. It can be seen that the temperature of the solution increases exponentially to reach a plateau corresponding to the thermodynamic equilibrium (steady state). As suggested in the literature [16,33,34], the transient temperature increase of NPs in solution can be described by an exponential variation with time. To extract information about the transient thermal state, we, therefore, used a formulation derived from this description and fitted the temperature data according to the following equation:

(1) $$∆T(t)=T_{\infty }\;(1-e^{-t/\tau })$$

where $T_{\infty }$ is the temperature increase related to the steady state and τ is the heating characteristic time. At τ, the temperature variation ΔT (τ) is equal to 63.2 % of the thermodynamic equilibrium temperature.

**Figure 3:**
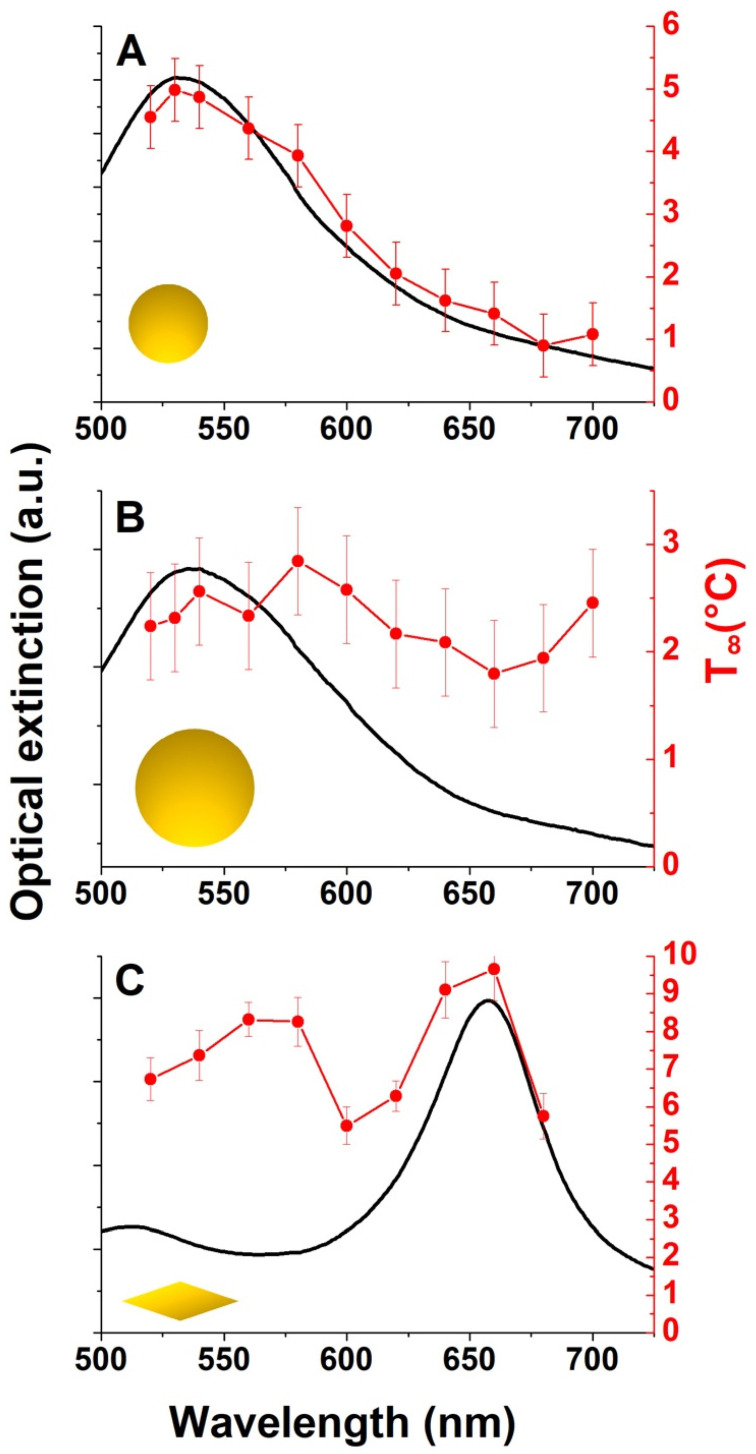
Extinction spectra (black line, left axis) and temperature increase $T_{\infty }$ (red circles, right axis) as a function of the excitation wavelength for the different solutions: AuNSs of 50 nm (**A**) and 100 nm (**B**) diameter and AuBPs of 45 nm length (**C**).

**Figure 4:**
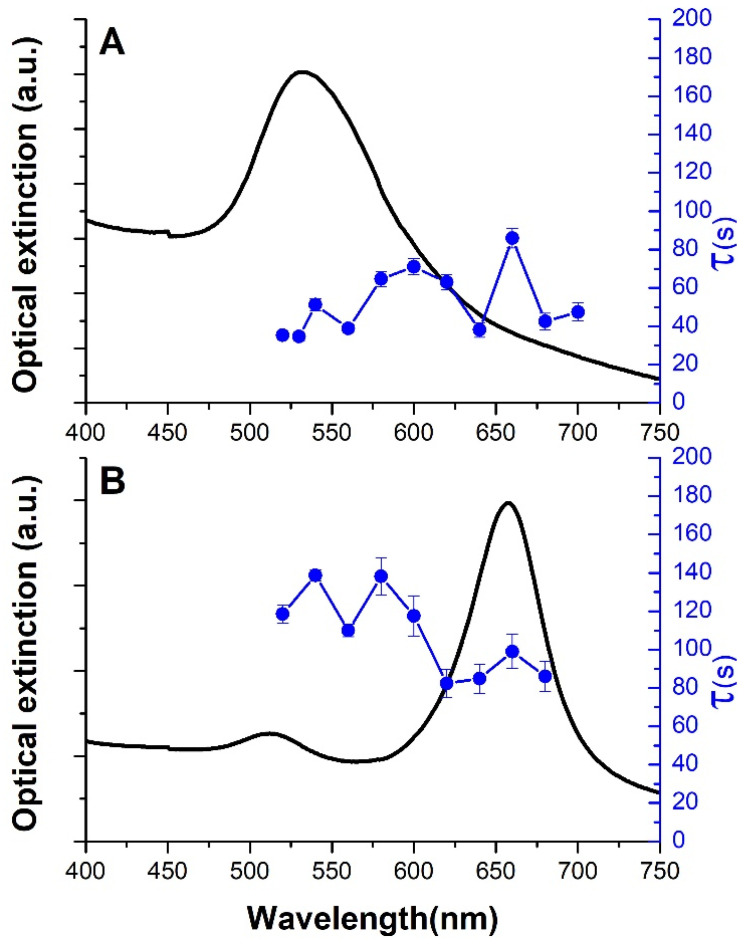
Extinction spectra (black curves, left axis) and τ constant (blue dots, right axis) as a function of the excitation wavelength for the 50 nm diameter AuNSs (**A**) and AuBPs (**B**). The blue lines between the blue dots are just a guide for the eyes and have no physical meaning.

Therefore, we fitted the temperature evolution using Equation (1) for each excitation wavelength and then plotted both parameters $T_{\infty }$ and τ as a function of the excitation wavelength (Figure 3 and Figure 4).

## 3. Results

As shown in Figure 3, the AuNSs exhibit LSPRs at 532 nm and 542 nm for diameters of 50 nm and 100 nm, respectively, whereas the AuBPs exhibit two LSPRs, at 515 nm (transversal mode) and 655 nm (longitudinal mode).

Let us first focus on the temperature increase observed for the AuNSs (Figure 3A,B). It can be observed that, in both cases (50 and 100 nm diameter), $T_{\infty }$ is not constant but varies by a few degrees Celsius depending on the excitation wavelength. Figure 3A shows that this temperature variation is directly related to the plasmon resonance of the 50 nm diameter AuNSs, whereas it is slightly red-shifted with respect to the plasmon resonance for larger particles (100 nm diameter) (Figure 3B).

Furthermore, it can be observed that the maximum temperature increase depends on the NP size, as expected [35]. The AuNSs reach $T_{\infty }$ of 5.0 °C for 50 nm and 2.8 °C for 100 nm diameter. These values are comparable to those already observed for similar structures, but under different excitation conditions as shown by Moustaoui et al. [20]. However, to gain insights into the role of NP geometry in the thermal process, we investigate the photothermal response of each type of NP. To achieve this, we assume the simultaneous excitation of a large number of nanoparticles evenly dispersed throughout the medium (colloidal NP in solution) within the laser beam (2 mm diameter at concentrations around 10 pM). Thus, we can consider this as a macroscale continuum distribution, as described by Pezzi et al. [36]. The thermal response within the solution can therefore be characterized as follows:

(2) $$∆T=2\pi \cdot w\cdot c(\lambda )\cdot n_{NP}\cdot I_{0}$$

with w, the laser beam radius, c(λ), the photo-heating effectiveness of the system, $n_{NP}$, the total number of NPs, and $I_{0}$, the average laser beam intensity.

The temperature variation is therefore proportional to the laser power and to the number of NPs in the solution (as shown experimentally in [20,21]). In our case, the radius of the laser, w, and $I_{0}$ remain constant for all experiments and measurements. The ratio between the temperature variation and the concentration must give us information on the photo-heating effectiveness, c(λ), and on its evolution according to the type and size of the NPs. We applied this calculation to the highest temperature, $T_{\infty }$, measured near the LSPR, and divided it by the concentration of NPs in the solution. We could then find the following $T_{\infty }$ per pM for both AuNSs samples: 0.336 °C/pM for 50 nm diameter and 0.384 °C/pM for 100 nm diameter. There is a slight increase in temperature as the AuNS diameter increases.

The relationship between the temperature increase and the LSPR can be explained by the change in the size of the NSs. A larger diameter means a larger volume and, therefore, an increased absolute absorption cross-section, which explains the aforementioned temperature increase.

In fact, the plasmon resonance contains both absorption and scattering contributions. Liu et al. [18] showed that NP heating is mainly related to their absorption cross-section. The photothermal efficiency, i.e., the fraction of the extinction energy converted into heat, is given by the ratio of the absorption cross-section to the scattering cross-section. This ratio changes as the size of the NPs is modified [24], as can be observed for the different NPs that we used (see Figures S2–S4). Baffou et al. [24] have shown that absorption dominates for AuNSs with diameters smaller than 90 nm, whereas scattering dominates for larger ones. Figure S2A shows the corresponding calculations of extinction, scattering, and absorption cross-sections using Mie theory. It can be seen that the absorption and scattering phenomena depend on the diameter of the AuNS. For the 50 nm diameter AuNS, the absorption cross-section dominates, showing a weak scattering (see Figures S2A and S3). This explains why the temperature evolution with excitation wavelength is in very good agreement with the LSPR profile, since the temperature increase is directly related to the absorption process (higher absorption, higher temperature increase). On the other hand, in the case of 100 nm diameter AuNSs, the scattering contribution is dominant in the extinction spectrum (see Figures S2B and S3). The temperature evolution then has a different profile compared to the LSPR extinction cross-section, since the scattering does not contribute to the temperature increase. Therefore, the observed temperature evolution cannot be perfectly correlated with the LSPR profile. However, even though the scattering is larger for 100 nm diameter AuNSs, the absorption is also larger for the 100 nm AuNSs compared to the 50 nm AuNSs. The temperature increase is then higher for the 100 nm AuNSs than for the 50 nm AuNSs. It is also clear that the temperature profile is slightly broader than the extinction spectrum in both cases. This can be explained in terms of sample irregularities: geometric polydispersity together with aggregation usually leads to a wider electromagnetic response.

When the NP morphology changes from nanosphere to bipyramid, the extinction spectrum shows two maxima, corresponding to the transversal and longitudinal plasmon modes (see Figure 3 and Figure S4 for more details on the electromagnetic response of bipyramids). This provides an opportunity to identify the contribution of each mode to the heating phenomenon. The transversal mode is located around 515 nm, while the longitudinal plasmon resonance appears at 655 nm.

Figure 3C shows that the temperature curve exhibits a similar profile to the extinction spectrum. $T_{\infty }$ has two maxima, like its extinction spectrum. While the strongest maximum corresponds to the wavelength of the longitudinal mode, the weakest one is slightly red-shifted with respect to the transversal mode. In particular, the AuBPs reach a maximum $T_{\infty }$ of 8.3 °C (0.072 °C/pM) and 9.7 °C (0.083 °C/pM) for the transversal and longitudinal modes, respectively. The temperature value per pM obtained for the longitudinal mode is slightly higher than that observed for the transversal mode. AuBPs have two sharp tips, which could induce a larger $T_{\infty },$ as already observed for nano-urchins [20]. Note that the transversal mode is almost as efficient as the longitudinal one, while the intensity of this mode in the extinction spectrum is much lower. To interpret this result, we performed some simulations on randomly distributed AuBPs in solution to get as close as possible to the experimental conditions (Figure S5). When the laser polarization is parallel to the long axis of the AuBPs (Figure S5b), the absorption cross-section of the transversal mode is largely lower than that of the longitudinal mode. On the other hand, when the AuBPs are randomly oriented (Figure S5a), some of them are misaligned with the laser polarization, leading to an enhancement of the transversal mode excitation. Therefore, their absorption cross-section is greatly increased. It can then be concluded that a greater amount of optical energy is absorbed by this mode, leading to more efficient heat generation, as observed experimentally. This effect can explain why the temperature increase for this mode is higher than the one expected from the extinction spectrum.

Moving on to the transient thermal analysis, we were able to estimate the heating characteristic time, τ, (Figure 4) by fitting the temperature increase data as a function of time (Figure 1C) using Equation (1). In both cases, τ is shorter in the LSPR region, thus highlighting that the heating process accelerates close to the resonance wavelength and when the $T_{\infty }$ reaches its highest values.

**Figure 5:**
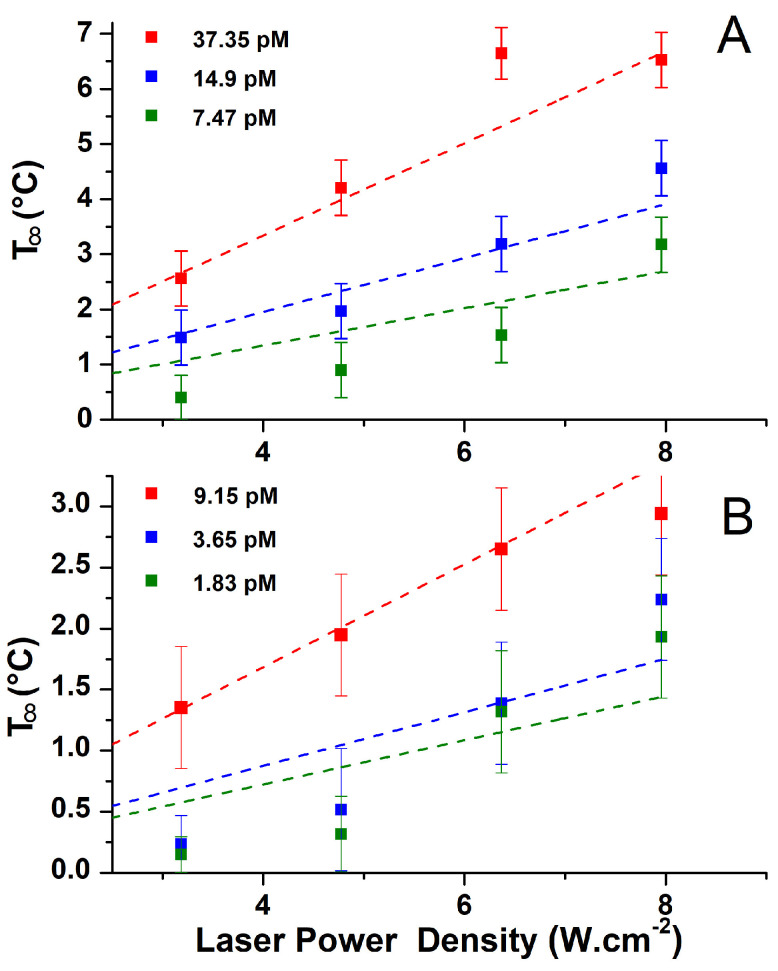
Temperature increase, $T_{\infty }$, measured with a 560 nm excitation wavelength for 50 nm (**A**) and 100 nm (**B**) diameter AuNSs as a function of excitation laser power for three different solution concentrations. The dotted lines correspond to the linear fit of the dots. The parameters of the fit are reported in Table S1.

The effect of the excitation power and AuNS concentration on the $T_{\infty }$ was also investigated. Figure 5 shows the temperature increase for three different AuNS concentrations (for both 50 and 100 nm diameters) as a function of the laser beam power. It can be seen that, for a given concentration, the temperature increase grows with the input power in all configurations. Indeed, Baffou et al. have shown that the heat dissipated by AuNPs is proportional to the absorbed power during laser illumination [3,24]. This power, Q, can, therefore, be approximated by the following relation:

(3) $$Q=\sigma_{abs}I$$

where $\sigma_{abs}$ is the absorption cross-section of the NP and I is the laser irradiance (power per unit area). As the laser power is increased, the power absorbed by the NPs increases proportionally (Table S1), and the heat generation is greater.

For each laser power, we also plotted the $T_{\infty }$ dependence for three different concentrations (Figures S6 and S7, Table S2). There, the value of $T_{\infty }$ for a zero concentration corresponds to the value of $T_{\infty }$ of the water for a given laser power. We can clearly see that the value of the temperature for the zero concentration rises as the laser power increases. For all laser powers, the $T_{\infty }$ exhibits a linear growth with the concentration. The increase in concentration results in a higher population of nanoparticles within the irradiated volume, thereby increasing the ability of the colloidal system to absorb input optical power from the incident light. Consequently, this enables a more pronounced temperature increase. This observation confirms that the $T_{\infty }$ corresponds to a macroscale continuum distribution.

## 4. Conclusions

Here, we investigated the relationship between the plasmon resonance of AuNPs and their photothermal response. We studied the heat generated by AuNPs in aqueous solutions (nanospheres and bipyramids at different concentrations) as a function of the illumination conditions, i.e., laser wavelength and power. We have shown that the heat generated is correlated with the plasmon resonance for 50 nm diameter nanospheres (maximum of heat generation for the LSPR position), where the absorption cross-section dominates the extinction spectrum. For a larger diameter, specifically 100 nm, the maximum temperature increase is red-shifted with respect to the plasmon resonance, as the extinction spectrum mainly reflects the dominant scattering contribution. In the case of bipyramids exhibiting both longitudinal and transversal plasmon modes, we observed a direct correlation between the heat generation yield and the longitudinal mode. In addition, the maximum temperature increase was red-shifted with respect to the transversal plasmon mode. In the latter case, we also observed that the longitudinal and transversal modes have the same heat generation efficiency due to the random distribution of the NPs in the solution. We also confirmed that the heating efficiency is proportional to the excitation power and to the NP concentration, confirming that we are in a state of a macroscale continuum distribution. Thus, the photothermal properties of the plasmonic NPs can be correlated with their optical and morphological properties, showing their dependence on the NP geometry and distribution as well as on the physical origin (absorption and scattering) of the light extinction.

## Data Availability

Data underlying the results presented in this paper are not publicly available at this time but may be obtained from the authors upon reasonable request.

## Supplementary Materials

The following supporting information can be downloaded at: https://www.mdpi.com/article/10.3390/nano14100820/s1, Figure S1: The experimental setup used to perform photothermal measurement; Figure S2: Extinction, scattering, and absorption cross-sections simulated using Mie theory, plotted versus the temperature elevation T_∞_ (red circles) measured for 50 nm (A) and 100 nm (B) diameter nanospheres; Figure S3: Scattering (a), absorption (b), and extinction (c) cross sections, and temperature increment (d), simulated using the Finite Element Method, for 25 nm and 50 nm radius spheres under circularly polarized light; Figure S4: Scattering (a), absorption (b), and extinction (c) cross-sections, and temperature increment (d), simulated using the Finite Element Method, for 45 nm and 50 nm length bipyramids under circularly polarized light; Figure S5: Scattering (blue line), absorption (red line), and normalized extinction (black line) cross-sections of three examples of colloids composed of six bipyramids randomly oriented within a water environment; Figure S6: Temperature increase of a 50 nm diameter nanospheres solution, excited using 560 nm laser wavelength, with different laser powers as a function of nanoparticle concentration; Figure S7: Temperature increase of a 100 nm diameter nanospheres solution, excited using 560 nm laser wavelength, with different laser power as a function of nanoparticle concentration; Table S1: Parameters of the linear fit of the temperature increase depending on the laser powers presented in Figure 5; Table S2: Parameters of the linear fit of the temperature increase depending on the nanosphere concentrations presented in Figures S6 and S7.
