# Supplementary material for: Correlation between Plasmonic and Thermal Properties of Metallic Nanoparticles
Source: Nanomaterials (Basel). 2024 May 7;14(10):820. doi: 10.3390/nano14100820 (PMC11123767; doi:10.3390/nano14100820)
Supplement: Supplementary file 1 [file nanomaterials-14-00820-s001.zip › nanomaterials-2942443-supplementary.pdf]

Supplementary Information

Correlation between Plasmonic and Thermal Properties of Metallic Nanoparticles

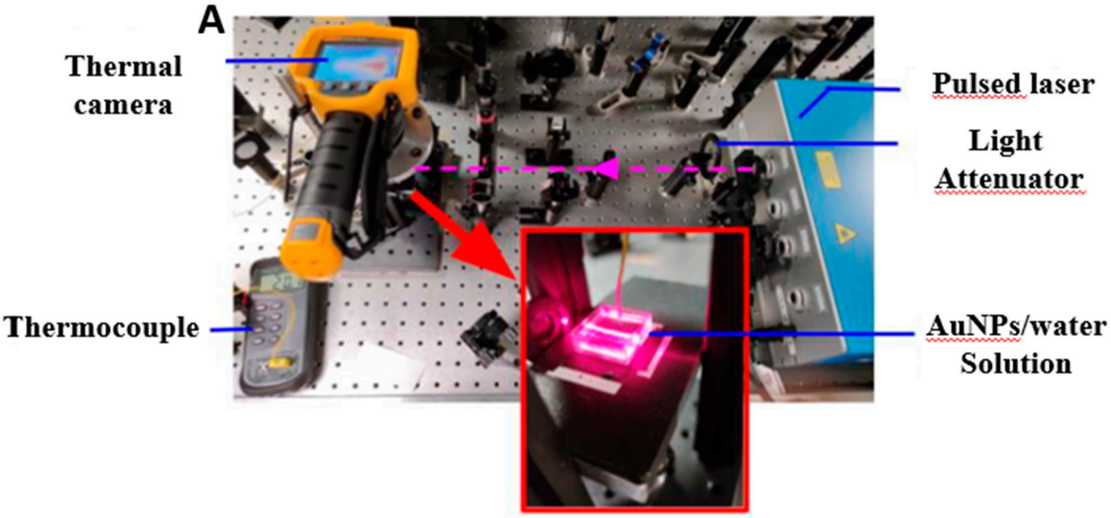

Figure S1: The experimental setup used to perform photo-thermal measurement.

Figure S1 shows the different elements used to measure the temperature of a gold nanoparticle solution. One can see, on the left, the thermal camera (TiR32 from Fluke) positioned just above the gold nanoparticle solution in order to record the temperature image of the whole solution from the top. One can observe the temperature image on the camera screen and the temperature gradient inside the solution (change of the color from blue to red). The camera is fixed on a metallic support to avoid any modification of its position and distance from the solution during all the photo-thermal experiments. On the right, one can see the output of the pulsed laser. The laser beam (not visible) is represented by the dashed pink line.

In this study, we wanted to excite the samples at specific wavelengths so that they had a nearly constant spectral gap between them (a gap of close to 20 nm between each excitation wavelength) across the visible range. We also needed a spectrally tunable light source to perform our experiments. The source that met our needs was a tunable pulsed laser: Inspire HF100 from Radiantis pumped by a MaiTai HP from Spectra Physics. We guarantee reproducibility of the measurement conditions by using the same pulsed laser source for all measurements.

Image inset in the red rectangle: This is a zoomed-in image of the gold nanoparticle solution that can be seen as it is placed below the camera and its support. The gold nanoparticle solution is in a plastic square vessel (see image inside the red rectangle). The solution is illuminated by the laser (red-lighted straight line in the vessel) that goes through the whole vessel length. At the top of this image, one can see the probe of the thermocouple (red wire), whose extremity is immersed inside the solution.

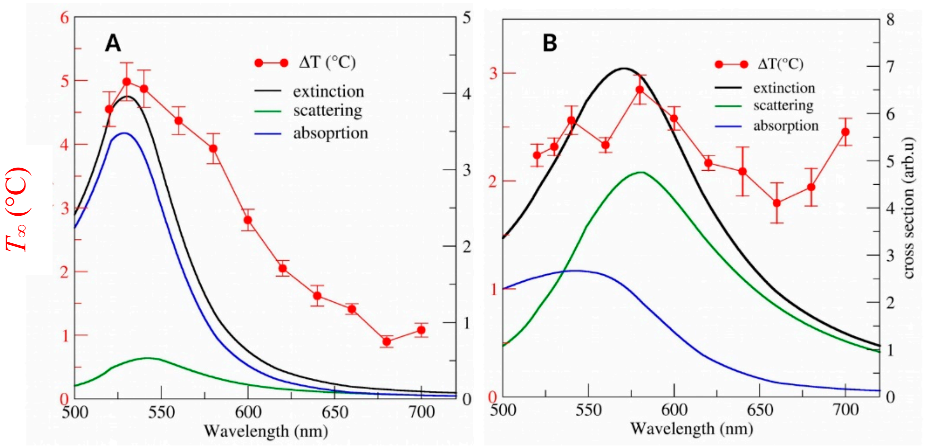

Figure S2: Extinction, scattering, and absorption cross-sections simulated using Mie theory, plotted versus the temperature elevation T_{∞} (red circles) measured for 50 nm (A) and 100 nm (B) diameter nanospheres.

Figure S2 shows the comparison of the temperature increase, T_{∞}, calculated with equation (1) of the main manuscript with the theoretical extinction, scattering, and absorption cross-sections of the nanoparticles. These three cross-sections were calculated using Mie theory (mieplot software accessible on the following website: http://philiplaven.com/mieplot.htm). One can observe that the plasmon resonance (maximum of the extinction spectrum) is red-shifted when the nanoparticle size increases, and that the relative contribution of the scattering increases for larger spherical nanoparticles whereas the relative contribution of absorption decreases.

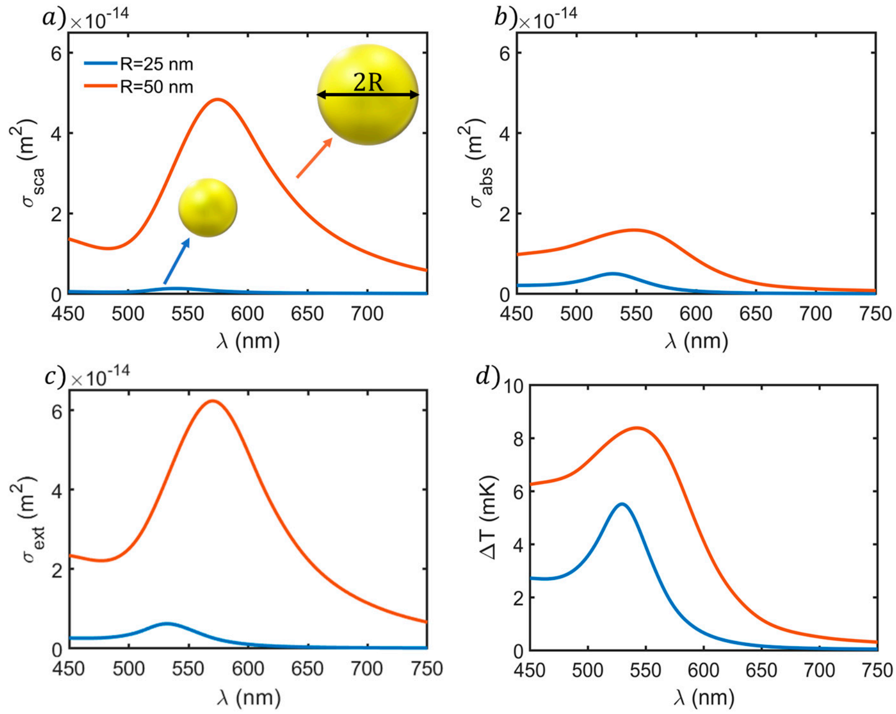

Figure S3. Scattering (a), absorption (b), and extinction (c) cross-sections, and temperature increment (d), simulated using the Finite Element Method, for 25 nm and 50 nm radius spheres under circularly polarized light.

Figure S3 shows the scattering (a), absorption (b), and extinction (c) cross-sections for two spheres with different nominal radii ($R = 25, 50\ nm$). It can be seen that, generally, the sphere response is red-shifted for the largest radius. This can be explained in terms of the mean free path of conduction electrons. For larger particles, electrons are free to move in a wider path due to being able to resonate for larger wavelengths (red-shift). This also increases the scattering and absorption, as seen in Figure S3a-c. Furthermore, the scattering response is red-shifted with respect to the absorption, as usual. Notice that the fraction of maximum absorbed/scattered energy is larger than that of the 25 nm radius sphere, while it is lower than that of the 50 nm radius sphere. This can be explained in terms of efficiencies: generally speaking, smaller particles are more efficient absorbers than larger ones, which are better scatterers. On the other hand, in Figure S3d, the temperature increases of the studied spheres (single particle temperature) are shown for an excitation power density of $I = 1.5 \times 10^{-4}$ mW/μm^2 (experimental value). As seen in this figure, the expected maximum temperature increases are about $\Delta T \approx 6\ mK$ and $\Delta T \approx 8\ mK$ for the 25 nm and 50 nm radius spheres. One can also observe that the maximum temperature increase is red-shifted for the 50 nm radius spheres, as observed experimentally (Figure 3).

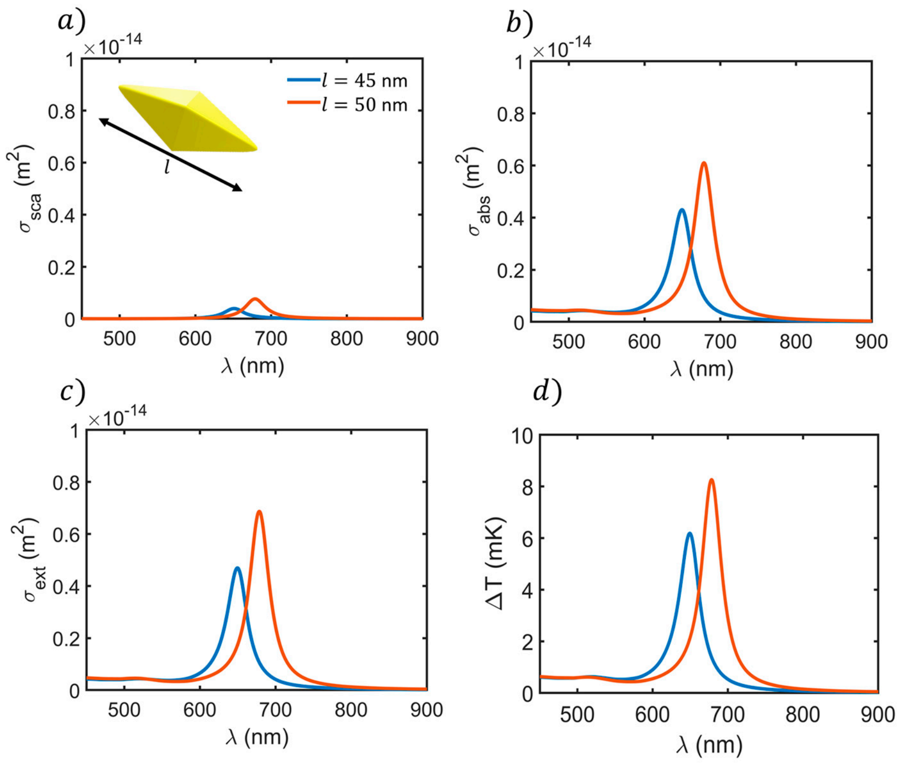

Figure S4: Scattering (a), absorption (b), and extinction (c) cross-sections, and temperature increment (d), simulated using the Finite Element Method, for 45 nm and 50 nm length bipyramids under circularly polarized light.

Figure S4 shows the scattering (a), absorption (b), and extinction (c) cross-sections for two bipyramids of 20 nm width and different nominal lengths ($l = 45, 50\ nm$). It can be seen that, generally, the largest bipyramid response is red-shifted with respect to the shortest case. This can be explained in terms of the mean free path of conduction electrons. For larger particles, electrons are free to move in a wider path and are thus able to resonate for larger wavelengths (red-shift). This also increases the scattering and absorption values, as seen in Figure S4. It is important to notice that both the absorption and scattering values present two peaks, corresponding to transversal and longitudinal modes of the bipyramid. As seen here, the transversal mode is much weaker than the longitudinal one. However, it is experimentally influential since the amount of absorbed and scattered energy will depend on the colloid density and orientation of particles with respect to the excitation polarization, so both modes will be enhanced or weakened depending on those experimental parameters. Thus, both modes are expected to be experimentally observed. On the other hand, in Figure S4d, the temperature increase of the studied pyramids (single particle temperature) is shown for an excitation power density of $I = 1.5 \times 10^{-4}$ mW/μm^2 (experimental value). As seen in this figure, the expected maximum temperature increases are $\Delta T \approx 6\ mK$ and $\Delta T \approx 8\ mK$ for the 45 nm and 50 nm length pyramids. It is worth mentioning that the value of the temperature increase strongly depends on parameters such as the input power density, the excitation time, or the particle volume, among others. Thus, different theoretical geometries can lead to slightly divergent temperature values for the same parameters, since they would contain different volumes.

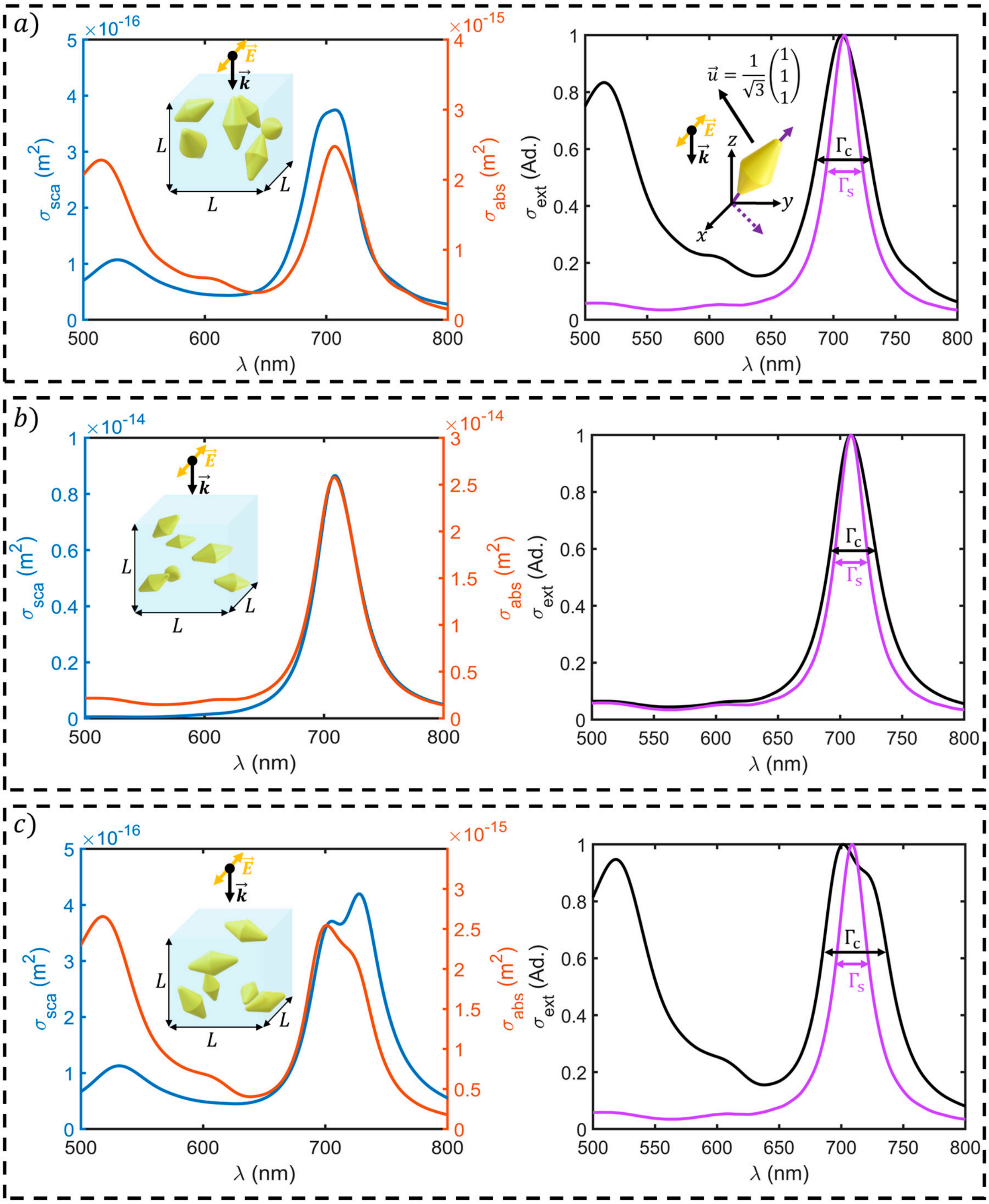

Figure S5. Scattering (blue line), absorption (red line), and normalized extinction (black line) cross-sections of three examples of colloids composed of six bipyramids randomly oriented within a water environment. Random distribution with particles' average orientations mostly misaligned (a), aligned (b), and partially aligned with the polarization direction (x-axis). In the left column, the red line corresponds to the absorption cross-section, and the blue line to the scattering cross-section. On the right column, the normalized extinction cross-section (black line) is compared with the normalized extinction of a single bipyramid oriented along the direction defined by $\vec{u} = (1,1,1)$ (purple line), as seen in the inset of the upper right subfigure. The simulation region is defined by a cube of side $L = 100\ nm$. Furthermore, Γ_c and Γ_s represent the spectral width of the collective and single longitudinal modes, respectively.

Once the effect of the collective alienation (one-dimension) on the optical response of bipyramids has been studied, a three-dimensional analysis is performed to illustrate the impact of collective effects on

the averaged optical response of colloids. Thus, in Figure S5, the scattering, absorption, and normalized extinction of three illustrative examples of simple colloids are shown. As seen in the left insets, the theoretical colloids are composed of six bipyramids randomly located and oriented. To evenly distribute the orientations, an equally distributed spherical set of points has been used, representing rotations. The maximum number of particles (six) is taken with regard to the maximum available computational resources (RAM memory).

In Figure S5a, the optical response of a colloid whose particles are mostly misaligned with the incident electric field orientation can be seen. Thus, most of the particles are totally misaligned with the electric field, and only one is mostly aligned with it. As a consequence, not only the longitudinal mode (most powerful) is excited, but also the transversal one. This explains the presence of both excitations in the normalized extinction.

A different feature can be observed in Figure S5b. Here, only the longitudinal mode is present and can be understood in terms of the set of orientations. In this second representative theoretical colloid, all particle orientations are contained in the plane of polarization, aligning the majority of them with the incident electric field. Therefore, the collective response is more similar to that of the single one. Indeed, it can be seen that both curves mostly match on the right side of Figure S5b.

Furthermore, a third case is analyzed in Figure S5c. As observed in the left inset, the particle orientations set in this theoretical colloid contain directions aligned and misaligned with the incident electric field, thus producing both excitations similarly. This is also a more compact colloid in which the interparticle distances are shorter. In fact, the scattering and absorption cross-sections reveal a splitting into two modes of the longitudinal response as a consequence of the proximity between particles (plasmon hybridization).

Additionally, the three examples demonstrate that, in general, the exposure of particles to the colloid neighborhood can produce not only a spectral shift but also a widening of the excited modes when they are electromagnetically excited. This can be seen on the right side of Figure S5, where $\Gamma_c > \Gamma_s$ for all cases. Regarding this spectral width, all examples present differences: the width of the most compact colloid (Figure S5c) is larger than the one presented in Figure S5a. Thus, the most ordered and non-compact system (Figure S5b) presents the shortest collective spectral width, which is the closest to the width of the single particle response.

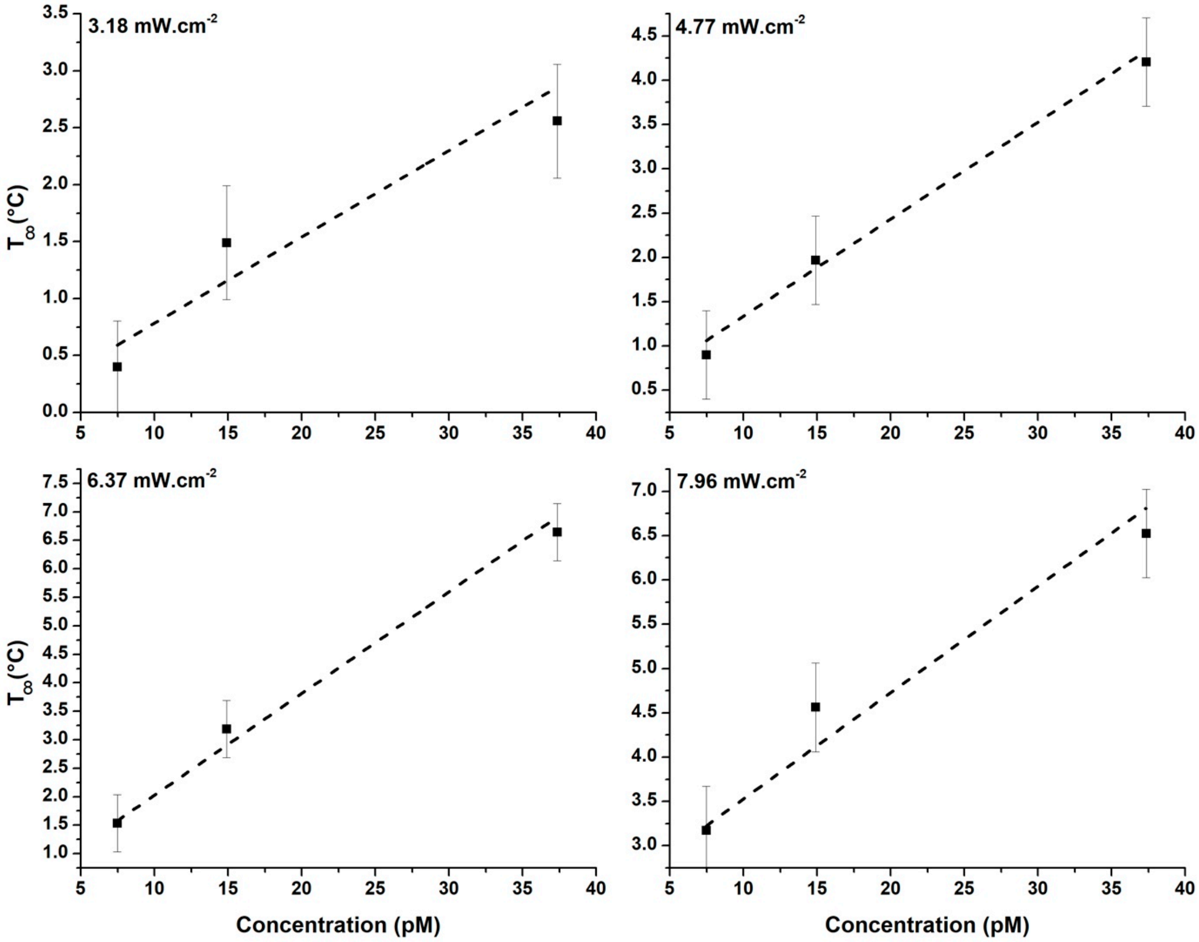

Figure S6: Temperature increase of a 50 nm diameter nanospheres solution excited using 560 nm laser wavelength with different laser powers (3.18, 4.77, 6.37, and 7.96 $W.cm^{-2}$), as a function of nanoparticle concentration. The zero-concentration temperature corresponds to the temperature increase of the water at the corresponding laser power.

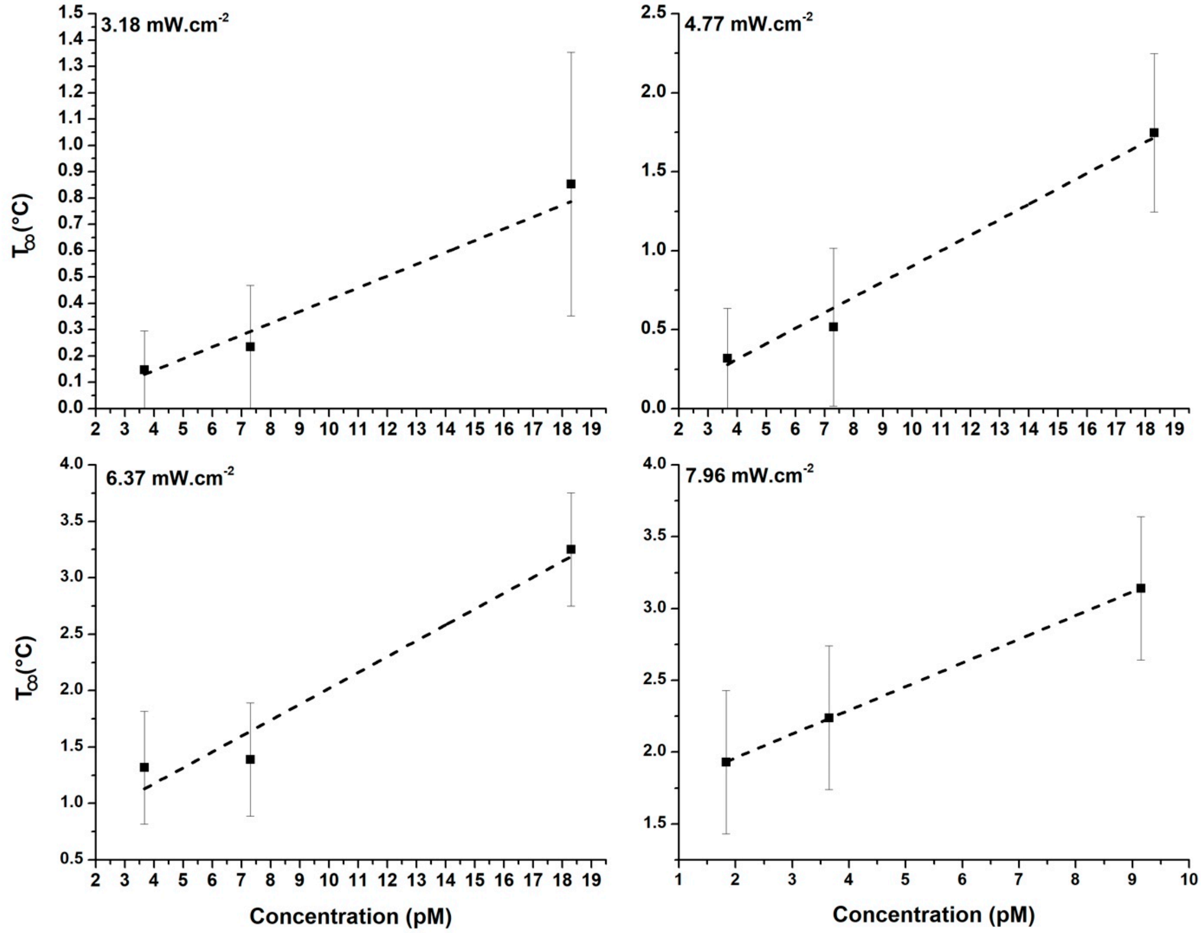

Figure S7: Temperature increase of 100 nm diameter nanospheres solution excited using 560 nm laser wavelength with different laser powers (3.18, 4.77, 6.37, and 7.96 W.cm^{-2}) as a function of nanoparticle concentration. The zero-concentration temperature corresponds to the temperature increase of the water at the corresponding laser excitation power.

The temperature increase, T_{∞}, increases linearly with the nanosphere concentration for both diameters and for all laser power densities. This reflects that the temperature increase has an extensive and cumulative behavior, meaning that the temperature increase corresponds to the simple sum of the heat contribution of all the nanoparticles inside the solution. Thus, no collective effect can be observed, as near-field nanoparticle coupling has a non-linear influence on heat generation.

Diameter (nm)	Concentration (pM)	a	b	R^2
50	7,47	0.55986	-1.4303	0.95251
	14,9	0.62066	-0.62146	0.95229
	37,35	0.85846	-0.07026	0.98304
100	3,66	0.37432	-1.0672	0.98761
	7,3	0.42001	-1.20725	0.97305
	18,3	0.25156	0.76275	0.91383

Table S1: Parameters of the linear fit of the temperature increase depending on the laser powers presented in Figure 5 (dotted lines). The linear fit formula is the following: $T_\infty = aP + b$ with P being the laser power. R^2 corresponds to the regression coefficient.

Diameter (nm)	Incident Power ($W.cm^{-2}$)	a	b	R^2
50	3,18	0.07584	0.02496	0.73315
	4,77	0.1095	0.24286	0.96739
	6,37	0.17856	0.24154	0.97388
	7,96	0.12	2.32937	0.90784
100	3.18	0.044845	-0.0381	0.89707
	4.77	0.09808	-0.07791	0.97446
	6.37	0.14067	0.14067	0.59853
	7.96	0.165	1.63229	0.99991

Table S2: Parameters of the linear fit of the temperature increase depending on the nanosphere concentrations presented in Figures S6 and S7 (dotted black lines). The linear fit formula is the following: $T_\infty = aC + b$ with C being the nanosphere concentration. R^2 corresponds to the regression coefficient.
